# Climate warming drives a temperate-zone lizard to its upper thermal limits, restricting activity, and increasing energetic costs

**DOI:** 10.1038/s41598-023-35087-7

**Published:** 2023-06-13

**Authors:** Lisa I. Doucette, Richard P. Duncan, William S. Osborne, Murray Evans, Arthur Georges, Bernd Gruber, Stephen D. Sarre

**Affiliations:** 1 Centre for Conservation Ecology and Genomics, Institute for Applied Ecology, University of Canberra, Bruce, ACT 2617 Australia; 2 Conservation Research, Environment and Planning Directorate, ACT Government, Mitchell, ACT 2911 Australia; 3 Department of Natural Resources and Renewables, 136 Exhibition Street, Kentville, NS B4N 4ES Canada

**Keywords:** Behavioural ecology, Ecology, Climate-change ecology

## Abstract

Lizards are considered vulnerable to climate change because many operate near their thermal maxima. Exposure to higher temperatures could reduce activity of these animals by forcing them to shelter in thermal refugia for prolonged periods to avoid exceeding lethal limits. While rising temperatures should reduce activity in tropical species, the situation is less clear for temperate-zone species where activity can be constrained by both low and high temperatures. Here, we measure the effects of natural variation in environmental temperatures on activity in a temperate grassland lizard and show that it is operating near its upper thermal limit in summer even when sheltering in thermal refuges. As air temperatures increased above 32 °C, lizard activity declined markedly as individuals sought refuge in cool microhabitats while still incurring substantial metabolic costs. We estimate that warming over the last two decades has required these lizards to increase their energy intake up to 40% to offset metabolic losses caused by rising temperatures. Our results show that recent increases in temperature are sufficient to exceed the thermal and metabolic limits of temperate-zone grassland lizards. Extended periods of high temperatures could place natural populations of ectotherms under significantly increased environmental stress and contribute to population declines and extinction.

## Introduction

Human induced climate change is occurring rapidly^1^ and challenging the ability of many species to respond in ways that they may have in the past^2^. Ectotherms are considered particularly vulnerable to the effects of climate change^3^ because of low tolerance to high temperatures and limited ability to regulate their thermal physiology^4^. For most ectotherms, even limited exposure to temperatures beyond their thermal maxima can be fatal^5,6^ forcing them to rely on altered behavioural thermoregulation to avoid overheating during the warmest times^4^. As global temperatures rise, more species may reach the limits of their ability to avoid critically high temperatures at key points in their life cycle, leading to population declines and extinctions^7–9^.

To counter high temperatures, ectotherms can alter their behaviour by seeking shelter in thermal refuges. However, spending more time in thermal refuges may come at a cost by decreasing the time available for other activities such as feeding and reproduction, potentially leading to lower rates of growth, survival and fecundity^2,10–12^ and increasing risks of local extinction^10,13^. These effects could be amplified because reduced food intake has been shown to lower optimal and maximum temperatures for growth, potentially causing a negative feedback loop whereby higher temperatures accelerate metabolic costs by reducing activity times, and consequently energy intake, which further lowers temperature tolerance^14^.

Lizards are considered to be among the ectotherms most vulnerable to climate warming, particularly in the tropics^15^. Tropical species already experience high temperatures, operate near their critical thermal limits^5,7,8,10^, and have greater niche specialization than lizards from higher latitudes^13,15,16^—Although montane tropical lizards may be an exception^17^. The impact of climate warming on temperate-zone lizards is less clear. Some lizards in the temperate-zone may be little affected by warming because they spend much of their time below their thermal optima^7,9^, with activity more likely restricted by low rather than high temperatures^4,18,19^. Climate warming could benefit lizards restricted by low temperatures by increasing their daily activity window, leading to fitness gains^18–22^. Alternatively, some temperate zone lizards could be vulnerable to climate warming if they operate in thermal environments close to key operational thresholds^23,24^ with little capacity to increase those thresholds^23^.

Studies predicting the effects of increasing temperature on lizards are often based on models that couple relationships between body temperature and performance, measured in the laboratory, with forecasted temperature changes used to predict likely outcomes in the field, often at broad spatial and temporal scales^13,18,22,25^. A difficulty with this approach is that models typically use predicted changes in overall temperature as input; an approach which may not reflect what happens in the field where lizards can regulate their temperature by adjusting the amount of time they spend in different thermal microhabitats^26–30^. Previous field studies on behavioural thermoregulation, involving individuals using refugia to stay within a preferred temperature range, have been largely limited to observations or intermittent measurements of lizard temperatures over several days^31–34^. Continuous quantitative data on the microhabitats occupied by lizards, coupled with fine scale temperature measurements within those microhabitats to assess thermal preferences, are rare^35–37^. This is because such data are particularly difficult to gather in the field, requiring intense study of individuals and their thermal landscape. Nevertheless, gathering such information is critical to evaluating the extent to which behavioural thermoregulation can mitigate the impact of increasing temperatures on individual and population-level performance^21,22,26,29,30,38,39^.

Here, we examine the potential impact of climate warming on a temperate-zone lizard by examining how a small, diurnal lizard, the endangered Canberra grassland earless dragon (*Tympanocryptis lineata*), uses thermal microhabitats to behaviourally regulate its temperature. *T. lineata* is a grassland specialist that is confronted with wide daily and seasonal temperature fluctuations, experiencing summer ground temperatures as high as 70 °C in exposed sites, and entering brumation in winter when temperatures fall below 0 °C. To escape temperature extremes, individuals commonly shelter in narrow, vertical burrows excavated in the soil by grassland arthropods, with burrows 1–2.4 cm wide and dug to a depth of 10–25 cm. These small burrows, along with the bases of dense tussock grasses, provide critical thermal refuges for the dragons. The small size of *T. lineata* (5–8 g; SVL 50–60 mm) makes them an ideal species to simultaneously record body temperature and environmental temperature using continuous temperature-sensitive radiotelemetry^40^. External temperature-sensitive transmitters and loggers have been shown to reliably measure skin temperatures (difference < 1 °C) for both ectotherms^35–37,41,42^ and endotherms^43–47^, and reliably predict body temperatures for small species (< 30 g) with differentials of less than < 2 °C^40,47^. Transmitters may heat or cool slightly faster than body temperature, but for an ectotherm less than 10 g the lag time between external and body temperature is less than a few minutes^40,48^.

In this study, we use temperature-sensitive transmitters to address two questions that have been difficult to quantify in the field: (1) To what extent are individuals able to alter their use of microhabitats to buffer themselves from temperature extremes? (2) At high temperatures, to what extent do energetic costs increase through a combination of restricted daily activity and higher resting body temperatures?

## Results

We attached temperature-sensitive transmitters to 28 adult *T. lineata* (15 males, 13 females) that were captured and released at four grassland sites in Canberra, Australia during the breeding season (austral spring–summer) over two consecutive years (Oct-Feb 2012/13 and 2013/14). Each animal was tracked for between 3 to 38 days (23 of these animals were tracked for > 5 days).

**Figure 1:**
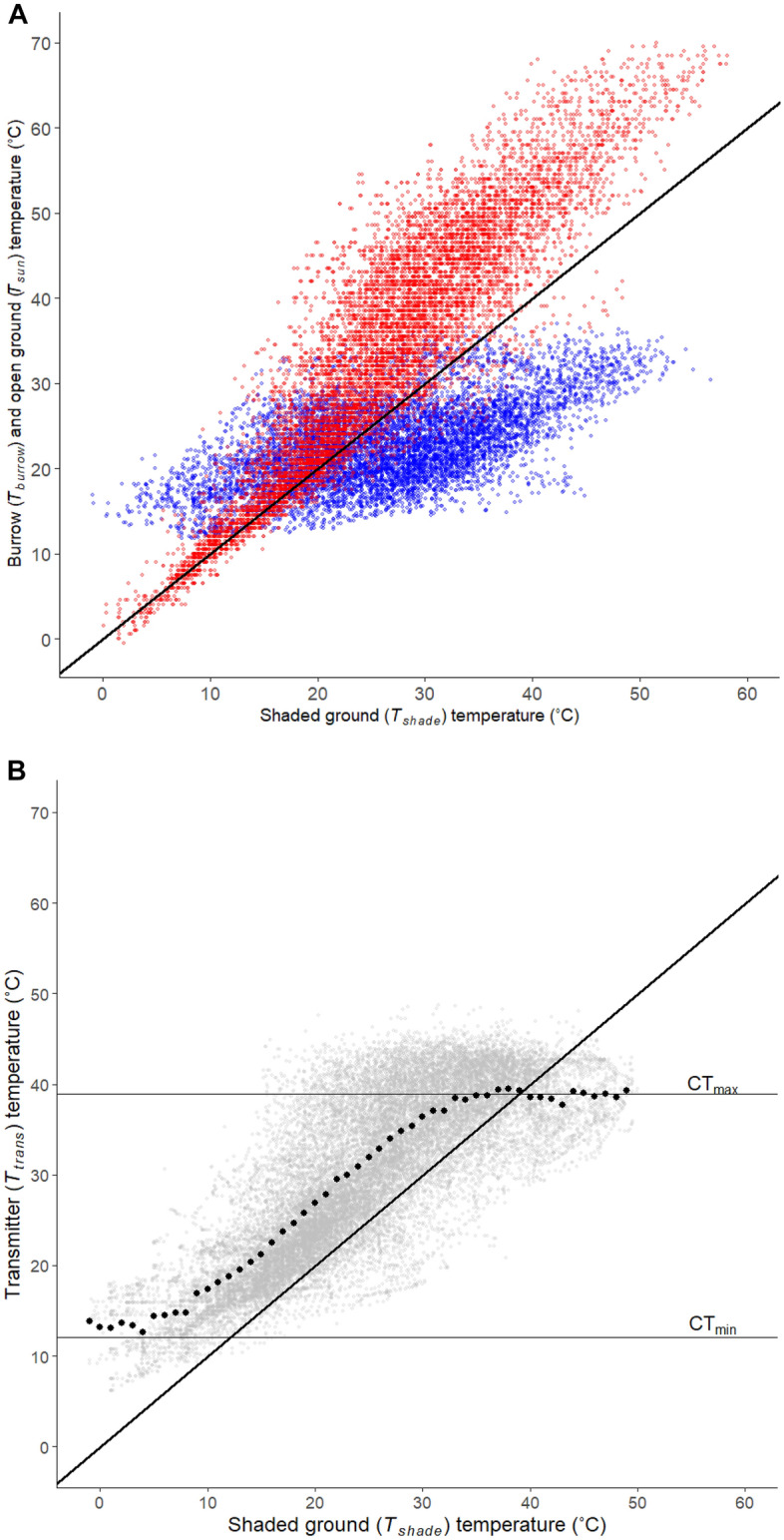
Environmental temperatures matched to dates, times, and locations of transmitter temperatures (T_trans_) for *T. lineata* recorded from Nov 2012 to Apr 2013 and Oct 2013 to Feb 2014. (**a**) Temperatures (36,059 measurements over 144 days) recorded in burrows (T_burrow_ = blue) and in copper pipe models placed in full sunlight (T_sun_ = red) in relation to shaded ground temperatures (T_shade_). The black line is a one-to-one comparison of shaded ground to other microhabitat temperatures. (**b**) *T. lineata* transmitter temperatures (T_trans_; 28 individuals, 144 days; 19,735 temperature measurements) plotted against shaded ground temperature. Grey circles are individual temperatures averaged across 90 s. Filled black circles are mean transmitter temperature for each degree of shaded ground temperature. The solid black line is the one-to-one line comparing T_trans_ to T_shade_. The horizontal lines show *T. lineata* body temperature (CT_max_ and CT_min_) at its upper and lower thermal limits (see “Methods”).

Laboratory tests on five female dragons placed in calibrated incubators at temperatures ranging from 15 to 40 °C showed that transmitter temperature (T_trans_) accurately measured dragon skin (T_skin_) and body temperature (T_body_) (T_skin_ = 0.924T_trans_-0.013, R^2^ = 0.939, P < 0.0001; T_body_ = 1.063T_trans_–3.20, R^2^ = 0.947, P < 0.0001; n = 705 measurements, N = 5 individuals). Transmitter temperatures also accurately measured T_skin_ and T_body_ under radiant heat at temperatures ranging from 20 to 35 °C (T_skin_ = 1.03T_trans_-3.68; R^2^ = 0.902; P < 0.0001; T_body_ = 1.05T_trans_-4.00T_trans_; R^2^ = 0.896; P < 0.0001). Nevertheless, there was a slight time lag such that T_skin_ and T_trans_ heated approximately 4 min faster than T_body_. Such a lag is expected for ectotherms weighing less than 10 g owing to the small size of the transmitters^40,48,49^. This faster heating of transmitters means that the T_trans_ values we report accurately reflect animal skin temperatures, but that body temperatures can lag behind. For this reason, T_trans_ values sometimes exceed the species critical thermal limit when animals spent short periods in full sunlight (Fig. 1b).

In the field, the transmitters recorded the temperature (T_trans_) of free-ranging individuals every 10 min for a total of 278 days and 19,735 transmitter measurements during daylight hours. At the same time, we measured microclimate temperatures in these grasslands using small temperature data loggers (iButtons). We used shaded ground temperature (T_shade_) as a baseline against which to compare temperature in two other microhabitats: exposed open ground (T_sun_) and the temperature in arthropod burrows (T_burrow_). Ground temperatures in the open sun can be extreme in these grasslands during late spring and summer, with an average daily maximum of 59 °C and temperatures sometimes exceeding 70 °C (Fig. 1a). Shaded ground temperatures were cooler (average daily maximum 38 °C), but even these could exceed 50 °C. Burrows, in contrast, provided a more thermally buffered environment (Fig. 1a): while shaded ground temperatures ranged from − 3 to 59 °C, burrow temperatures ranged from 12 to 36.5 °C (mean 23.2 °C). Laboratory tests showed a close correlation between T_trans_ and microclimate measures at ambient temperatures (T_shade and_ T_burrow_) in incubators (T_iButton_ = 1.01T_trans_–2.21, R^2^ = 0.967, P < 0.0001) and under solar radiant heat (T_sun_; Paired *T* test = 10.67; Two-tailed P < 0.0001). Shuttling between microhabitats allowed individuals to stay between the critical maximum (T_skin_ 38.3 to 42.8 °C, mean = 40.6 ± 0.8 °C; T_body_ 37.8 to 40.2 °C; mean = 38.9 ± 0.3 °C) and minimum (T_skin_ 8.3 to 11.5 °C, mean = 9.9 ± 0.8 °C; T_body_ 10.3 to 13.4 °C; mean = 12.1 ± 0.4 °C) skin and body temperatures recorded in laboratory experiments (Fig. 1b).

**Figure 2:**
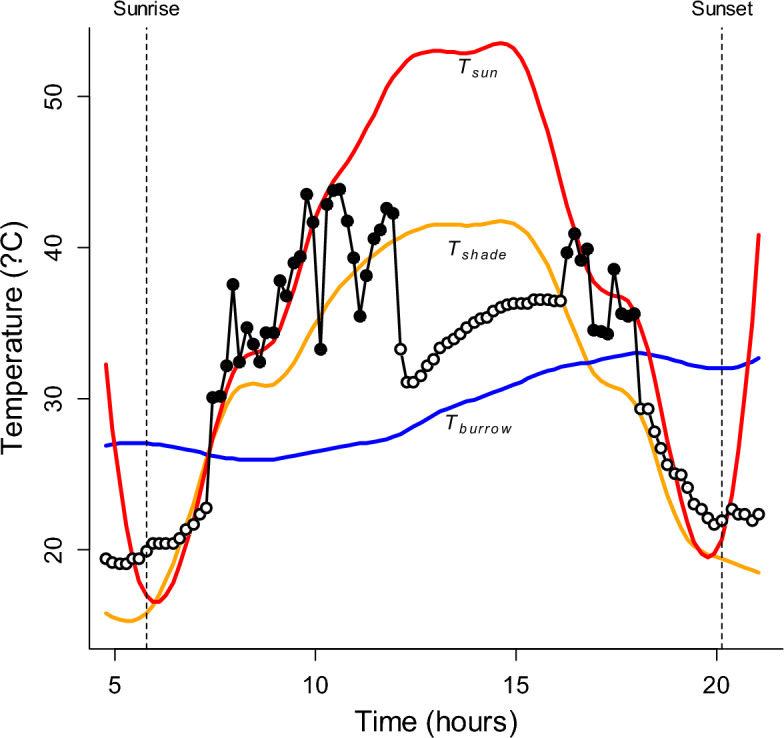
Plot of *T. lineata* transmitter temperatures (T_trans_) taken at 10 min intervals (circles) for one individual on 1 day, and microhabitat temperatures (T_sun_ = red line, T_shade_ = orange line, and T_burrow_ = blue line) at the same location for the same period. Filled circles indicate times when the individual was classified as active; open circles indicate times when the individual was classified as inactive. Dotted vertical lines show the times of sunrise and sunset. The dragon is in a warm refuge prior to sunrise, as indicated by T_trans_ being greater than both T_sun_ and T_shade_, and remains inactive until ~ 1.5 h after sunrise when T_trans_ starts to track T_sun_, indicating the dragon has moved into the open where it remains for over 2 hours. As T_sun_ rises above about 40 °C, the dragon starts to shuttle between open and shaded sites before moving into a cool refuge (T_trans_ < T_shade_) in the middle of the day where it remains inactive for several hours. It emerges again for a period in the late afternoon before becoming inactive again at around 1800 h, where T_trans_ starts to fall steadily in parallel with falling ambient temperatures (T_sun_ and T_shade_), with T_trans_ levelling out around sunset at a value greater than T_sun_ and T_shade_, indicating the dragon was in a warmer refuge for the night. Note, that T_burrow_ is the temperature at 150–200 mm depth in one burrow at the site, but the dragon could have taken refuge in another burrow and at a different depth, such that we don’t expect T_trans_ to track T_burrow_ precisely when the dragon is sheltering in a burrow.

**Figure 3:**
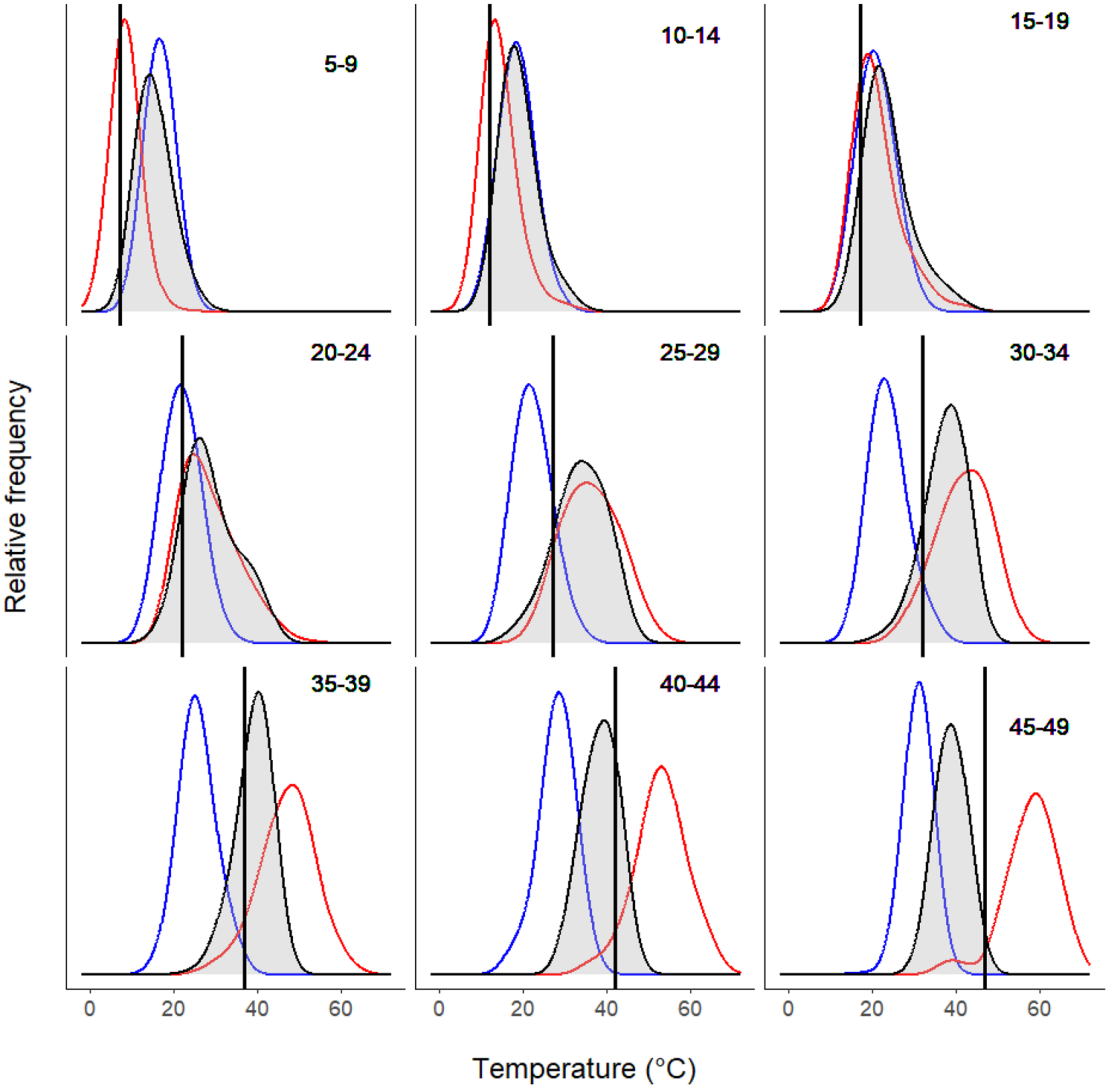
Frequency distribution of transmitter and microhabitat temperatures as a function of shaded ground temperature grouped into 5 °C bands (indicated by the numbers at the top right of each panel). The mean shaded-ground temperature (T_shade_) for each band is shown as the black vertical line. Blue shows the distribution of burrow temperatures (T_burrow_), red the distribution of full sun temperatures (T_sun_), and black line/gray shading the distribution of transmitter temperatures (T_trans_) for each band of shaded-ground temperature.

Plotting dragon transmitter temperature (T_trans_) as a function of shaded ground temperature (Fig. 1b) revealed how dragons altered their use of thermal microhabitats as shaded ground temperature changed. Points above the 1:1 line were transmitters that were warmer than shaded ground, indicating dragons were in more open microhabitats, while points below the line indicate that dragons were occupying cooler microhabitats such as burrows. Across the range of shaded ground temperatures (− 3 to 59 °C), average T_trans_ remained within the thermal tolerance limits of the species (Fig. 1b) because individuals adjusted the amount of time they spent in different thermal microhabitats (Fig. 2). For example, when shaded ground temperature fell below the lower critical thermal limit for the species, average transmitter temperature remained above this limit because dragons spent more time in thermally buffered sites, such as burrows, particularly at night and during the coolest parts of the day (Fig. 2). As evidence for this, the distribution of transmitter temperatures closely matched the distribution of burrow temperatures when shaded ground temperature was less than 15 °C (Fig. 3). At these temperatures, burrows were generally warmer than both shaded and open ground sites (Figs. 1a, 3).

At shaded ground temperatures between 15 and 25 °C, the distribution of transmitter temperatures closely matched the distribution of open ground (T_sun_) temperatures (Fig. 3), implying that dragons spent much of the time using open sites in this temperature range (see Fig. 2). As shaded ground temperature rose above 25 °C, open ground temperatures steadily exceeded transmitter temperatures, implying that dragons increasingly avoided warmer open sunny sites at higher temperatures (Fig. 3). At shaded ground temperatures greater than 40 °C, most transmitter temperatures were below this value, implying that dragons were avoiding shaded ground sites in favour of cooler microhabitats, such as burrows. The point at which shaded ground temperatures were sufficiently warm that dragons began to avoid this microhabitat is indicated by the sharp inflection in the average transmitter temperature curve when shaded ground temperature approached the upper thermal tolerance limit of the species (Fig. 1b). At shaded ground temperatures above about 35 °C, average transmitter temperature remained relatively constant, and below the thermal tolerance limit, as dragons increasingly sought refuge in cooler microhabitats. These data show that dragons sought refuge in more thermally buffered environments, such as burrows, at both high and low shaded ground temperatures to maintain average temperature within their thermal limits. As such, key changes in the use of microhabitats in the field coincided with the upper and lower critical temperature thresholds of this species.

**Figure 4:**
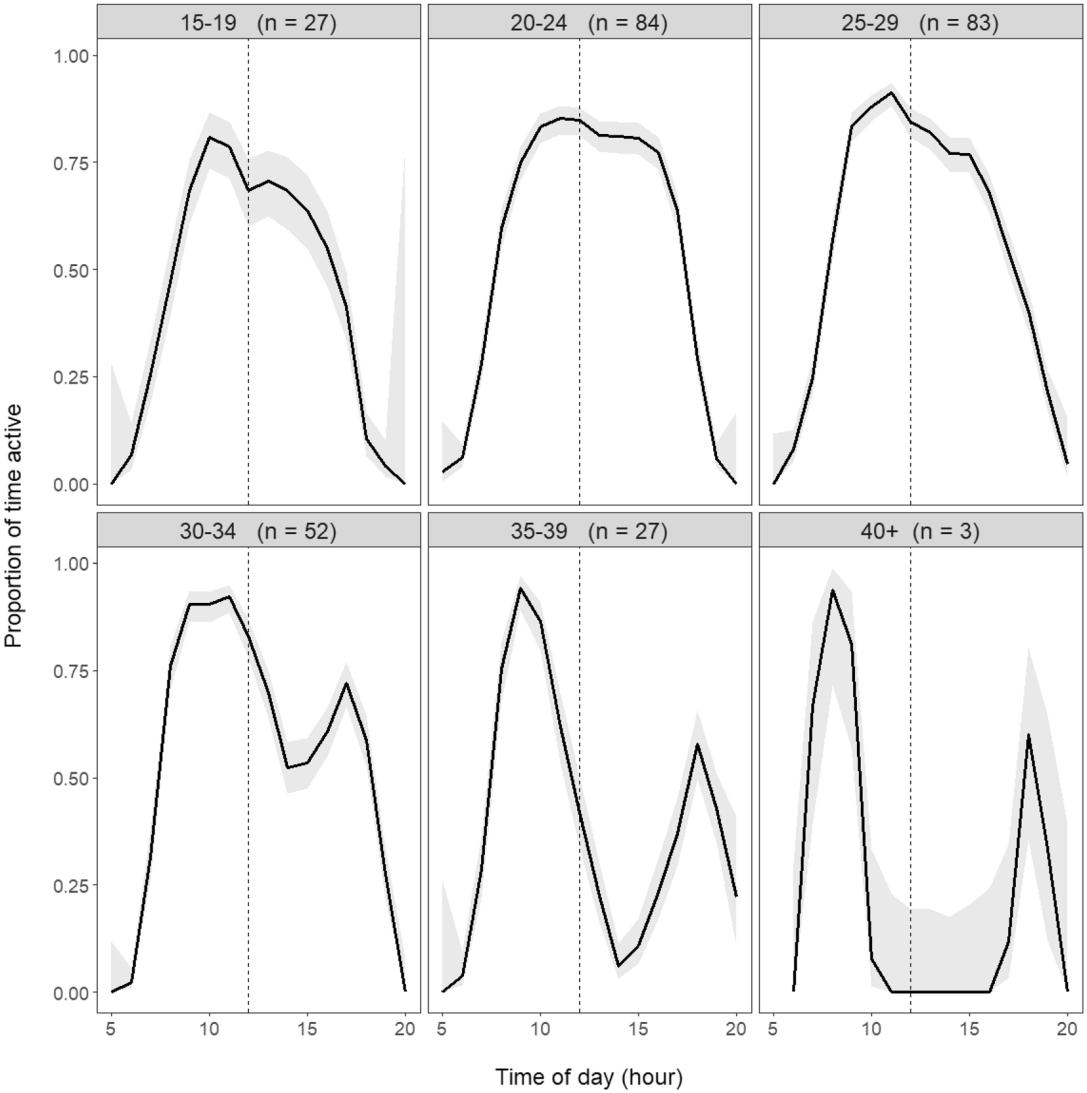
Proportion of time for each hour of the day between sunrise and sunset that *T. lineata* were classed as active, with the days grouped by maximum daily air temperature into 5 °C bands (indicated at the top of each panel, along with the number of lizard days, *n*, in each group). As maximum daily air temperature rises above 30 °C, there is an increasingly marked period of inactivity around the middle of the day as dragons escaped the heat by sheltering in thermal refuges. Grey shading shows 95% confidence intervals (CI) for the proportion of time active. The increase in the 95% CIs at the start and end of photoperiods reflect the smaller number of observations and hence larger uncertainty as photoperiod length differed over time.

**Figure 5:**
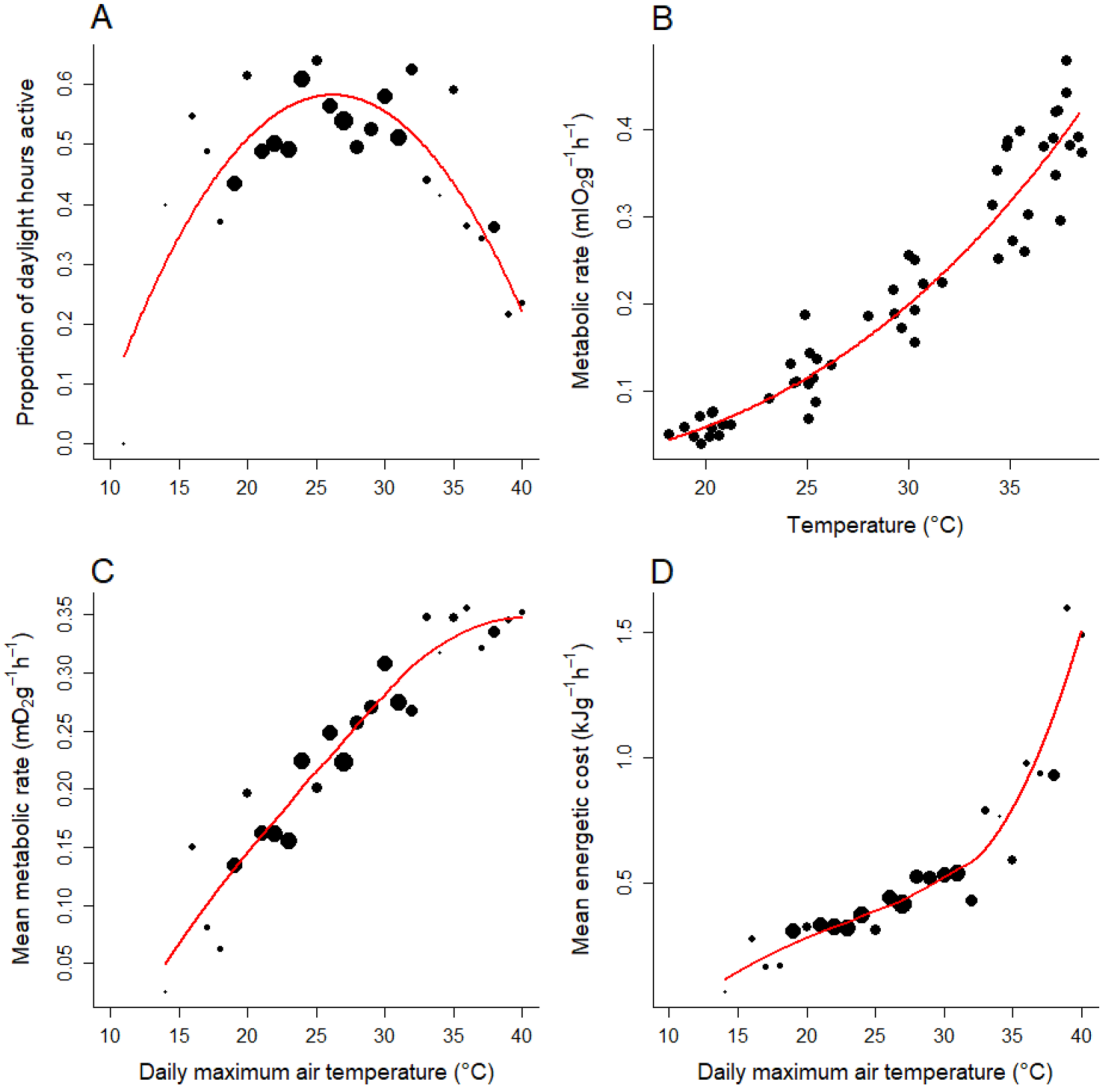
(**A**) The relationship between maximum daily air temperature and proportion of daylight hours active. The solid red line is a quadratic regression model fitted to the data, for which both the linear and quadratic terms were significant (P < 0.0001, R^2^ = 0.71). (**B**) The relationship between resting metabolic rate and temperature for *T. lineata* measured in the laboratory ($\text{Metabolic rate}= {e}^{-11.7+2.99*\mathrm{log}(\text{Temperature)}}$, P < 0.0001, n = 12 dragons). (**C**) The relationship between maximum daily air temperature and mean hourly resting metabolic rate, with a LOESS function (red line) fitted to indicate the trend. (**D**) The relationship between maximum daily air temperature and mean energetic cost during each day (mean hourly resting metabolic rate/proportion of daylight hours active), with a LOESS function (red line) fitted to indicate the trend. In panels (**A**), (**C**) and (**D**), each filled circle represents 1 day, with the size of the circle proportional to the number of dragons measured on that day (total of 278 dragon days).

Essential activities, such as feeding and finding mates, require dragons to be above-ground^50^, but high and low above-ground temperatures force dragons into thermal refuges such as burrows. Seeking refuge is a well-known behaviour used by lizards to escape high temperatures^22,30,51^. However, we lack field data quantifying the extent to which high temperatures curtail above-ground activity, and hence the impact that high temperatures may have on the animal’s ability to forage above-ground to support their energetic demands. To understand the impact of a shortened daily activity window caused by high temperatures, we classified transmitter temperatures according to whether dragons were above or below shaded ground and burrow temperature for each transmitter temperature record. Transmitter temperatures below shaded ground temperature indicate the dragon was using a thermal refuge to escape heat, while transmitter temperatures above shaded ground temperature, but at or below burrow temperature, indicate that the dragon was using a refuge to escape cold. During daylight hours, above-ground activity was curtailed in the early morning and late evening as dragons sought refuge from cooler above-ground temperatures, particularly on days when the daily maximum air temperature was low (Fig. 4). On days when the maximum air temperature rose above 30 °C, dragons increasingly sought thermal refuge from high temperatures during the middle of the day (Figs. 2, 4). This resulted in a quadratic relationship between daily activity (the proportion of daylight hours dragons were active above-ground) and maximum daily air temperature (Fig. 5A). Dragons spent, on average, more than half the day active when daily maximum air temperatures were between about 20–32 °C. As the daily maximum temperature rose or fell beyond these values, dragons spent an increasing proportion of the day in thermal refuges. On days when the maximum air temperature approached 40 °C, dragons spent about 80% of daylight hours in thermal refuges to avoid the heat of more open environments and to maintain their temperature within thermal tolerance limits.

To assess the implications of a shortened daily activity window on dragon energetics, we measured resting metabolic rates (RMR) for 12 post-absorptive individual *T. lineata* (6 males, 6 females) at five temperatures (20, 25, 30, 35 and 38 °C) using flow-through respirometry^52,53^. Mean mass-specific RMR increased exponentially with temperature (Fig. 5B; $\text{Metabolic rate}= {e}^{-11.7+2.99*\mathrm{log}(\text{Temperature)}}$) and did not differ between sexes (P = 0.79). Resting metabolic rates (RMR in ml O_2_ g^–1^ h^–1^) were then used to calculate the average amount of energy expended by a dragon at rest in the field based on the recorded transmitter temperatures averaged on an hourly basis. We calculated the mean RMR of a dragon for each day that we had transmitter records by averaging the hourly RMR values and then plotted the daily mean as a function of maximum daily air temperature (Fig. 5C). The mean RMR increased as daily maximum air temperature rose, as expected for an ectotherm, but started to level out once daily maximum temperature exceeded about 32 °C. This is likely to occur because dragons used thermal refuges to escape the heat on warmer days (Figs. 1b, 3, 4) and thus limit their metabolic losses. Consequently, the mean RMR divided by the number of hours active increased sharply on days when the maximum temperature rose above 32 °C (Fig. 5D). We use the term energetic cost for the ratio: mean RMR/number of hours active. This ratio measures the amount of energy dragons must obtain per active hour by feeding to balance their daytime resting metabolic losses. While metabolic losses would also occur at night, dragons experienced lower temperatures overnight and almost always sheltered in cool microhabitats (Figs. 1 and 3), meaning nocturnal metabolic losses would be lower.

The resting energetic cost, and hence the energy dragons must obtain while active to offset this cost, increased about three-fold as daily maximum air temperature rose from 32 to 40 °C (Fig. 5D). Hence, while activity time was reduced on days with both low and high maximum temperatures, dragons incurred a substantially higher energetic cost on hot days because of greater metabolic losses at high temperatures coupled with reduced activity time in which to recoup those losses. Confining our calculations to daylight hours only and using resting metabolic rate as our measure of energy expenditure provides a conservative measure of total daily energy expenditure. If dragons are active, rates of energy metabolism are typically 1.5–3 times resting levels^54,55^.

**Figure 6:**
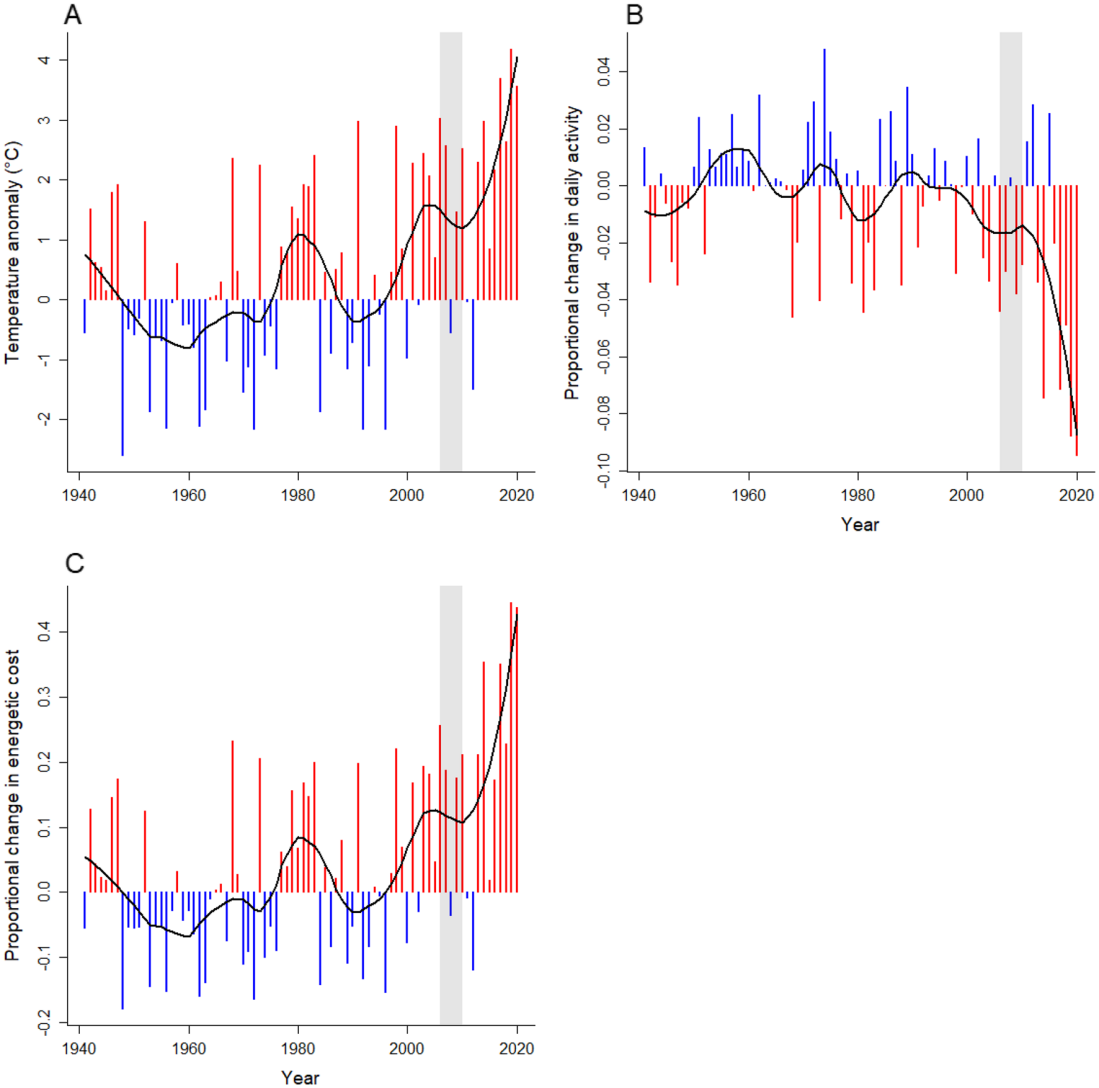
Estimated proportional change in activity and mean energetic costs for grassland earless dragons arising from changes in mean maximum daily air temperature for the years 1941–2020. (**A**) Mean maximum daily air temperature per year measured at the Canberra Airport for the period 1941–2020 and expressed as an anomaly from the mean for the period 1960–1990. (**B**) The mean proportion of time dragons were active during daylight hours (activity window) for each year expressed as a proportion of the mean for the period 1960–1990. (**C**) The mean energetic cost per year (mean hourly resting metabolic rate/proportion of daylight hours active) expressed as a proportion of the mean for the period 1960–1990. In each panel, blue columns indicate years below the 1960–1990 mean; red columns indicate years above the 1960–1990 mean; and the black line is a LOESS function fitted through the yearly values with span = 0.3. The grey shaded area covers the years 2006–2010 when the *T. lineata* population appeared to collapse.

To examine changes in the energetic cost over time, we gathered data on daily maximum air temperature during summer (December-February) for the years 1941–2020 recorded at Canberra Airport, the nearest climate station to our study sites (all sites were within 7 km of the climate station and at the same altitude). For each day during this period, we estimated the proportion of daylight hours dragons were active and the mean energetic cost based on the maximum daily temperature and the relationships shown in Fig. 5. We then calculated the mean maximum temperature, the mean daily activity time, and the mean energetic cost per day during the breeding season for each year (expressed as an anomaly from the mean for the period 1960–1990). Mean daily maximum temperature fluctuated around the mean from 1941 to 2000 but has increased in the period 2000 to 2020 to be on average more than 3 °C greater than the mean (Fig. 6A), resulting in a decline in mean daily activity during summer of up to 8% (Fig. 6B). The combination of hotter days and a shorter activity window caused the mean energetic cost for individual dragons to increase by up to 40% during the same period (Fig. 6C). This implies that between 2000 and 2020, increasing maximum daytime temperatures required dragons to increase their energy intake during their active period by up to 40%, relative to the 1960–1990 mean, to offset metabolic losses.

## Discussion

Ectothermic activity in temperate regions is usually constrained by both low and high temperatures, meaning it is unclear whether warming will be beneficial (increase the activity window) or detrimental (decrease the activity window) to temperate-zone populations. We show that during the critical spring–summer months of the breeding season the activity window declines sharply for *T. lineata* as daily maximum temperature rises above 32 °C, implying climate warming that resulted in a greater number of hotter days could negatively affect populations by forcing dragons to spend more time in thermal refuges, reducing the time available for essential activities such as foraging and finding mates.

On hotter days, reduced activity coupled with greater metabolic losses incurs a substantial energetic cost. Between the years 2000–2020, we estimate that dragons had to increase their energy intake during their daily active period by up to 40% to offset metabolic losses owing to rising temperatures, even after accounting for behavioral adjustments to limit those losses by using thermal refuges. This estimate is conservative because we have used resting metabolic rate as our measure of energy expenditure and confined our calculations to daylight hours only. *T. lineata* will be active for significant portions of the day (not just resting) and will range widely to forage^56^. Given that activity will increase energy expenditure beyond that indicated by resting metabolic rate, and that dragons will continue to expend energy at night but cannot forage, the energetic impact of the observed increase in maximum daily temperature over the last 20 years (Fig. 5) will be even higher than our analysis reveals. Populations of *T*. *lineata* suffered widespread collapse linked to the Millenium Drought between 2006 and 2010^57^. Our results suggest it is possible that an increase in the number of hot days in Canberra, with a sustained increase commencing around the year 2000 (Fig. 6A), could have played a role in this population collapse by reducing activity times (Fig. 6B) and increasing metabolic costs (Fig. 6C) that may have been difficult to recoup.

Sinervo et al.^2^ speculated that extinctions of lizard populations are likely if activity is restricted by 7 h or more per day, particularly during critical reproductive months. Our results show that *T. lineata* would exceed this threshold when daily maximum air temperatures rise above 35 °C (Figs. 3 and 4a) in the breeding season (October to February) when average day length is 13.5 h and *T. lineata* are active for less than 5.5 h. This is close to the daily maximum temperature where metabolic costs begin to rise sharply (Fig. 5D), suggesting the 7 h activity restriction roughly corresponds to a critical threshold in energy expenditure for *T*. *lineata*.

The microclimate temperatures we recorded in direct sunlight were up to 30 °C higher than those obtained previously using an inanimate lizard model in temperate Australia^18^, suggesting that ectotherms at temperate-zone sites can reach dangerously high temperatures in open environments and may not be able to maintain optimal temperatures (30–35 °C) in shaded environments^18,22^. In the scenarios modelled previously, lizards in full sun achieved body temperatures of around 40 °C, and thermoregulated by shuttling between sun and shade, maintained body temperatures of up to 33 °C^18^. Our results for *T. lineata* contrast sharply with these values, indicating that shaded microhabitats alone are insufficient to keep temperatures below critical thermal thresholds and that more thermally buffered refuges, such as burrows, are often required for survival (Figs. 1 and 2). Grasslands are exposed environments with shade only available close to the ground, or below ground in burrows during periods of low vegetation cover, such as in droughts. Consequently, grassland lizards will be exposed to radiant and conductive heat at ground level that far exceeds the shaded temperatures 1.2 m above ground at which air temperatures are measured. It is therefore likely that lizards are exposed to much higher temperatures in grasslands, for a given air temperature, relative to more heavily vegetated environments with canopy shade.

At warmer temperatures, we observed that individual *T. lineata* remained active by shuttling between open and shaded microhabitats, often moving into burrows to cool (Figs. 1, 2 and 3). This shuttling behaviour is believed to be the key to avoiding temperature extremes and surviving climate warming in temperate environments^8,18,19^ but has rarely been measured directly using body temperatures. The shuttling behavior we observed provides considerable capacity for *T. lineata* to manipulate body temperature, allowing dragons to remain above-ground on warm but not excessively hot days. However, this behavior depends on the availability of cooler microhabitats such as heavily shaded ground and burrows. Consequently, *T. lineata* is susceptible to the loss of these refuge habitats, including the removal of above-ground vegetation by burning, drought or overgrazing, and the loss of invertebrate species responsible for digging the burrows that dragons rely on for shelter^56,57^. Vegetation loss associated with drought, in addition to high temperatures, may have also contributed to the widespread population collapse in *T. lineata* from 2006 to 2010^57^. While it is possible that climate warming will extend the seasonal activity window for *T. lineata,* thereby shifting some activities to different times of the year, the extreme temperatures now experienced by these dragons in thermal refuges such as burrows (max 36.5 °C) suggests there may no longer be safe refuges on extreme days.

Our data offer strong empirical support for the proposition that high summer temperatures caused by climate warming may repeatedly exceed the thermal and metabolic limits of this species. If this were to also be the case for other temperate zone lizards, extended periods of high temperatures and reduced activity times over longer periods could place natural populations of lizards under significantly increased levels of environmental stress and contribute to population decline and local extinction. Vulnerability to warming will be a function of the thermal requirements of a species, their ability to adapt both physiologically and behaviourally to increased temperatures^58^, and the availability of thermal refugia. Consequently, habitat degradation that alters the availability of thermal refugia will interact with climate warming to further imperil populations by reducing the opportunity for individuals to escape extreme heat while simultaneously increasing the need to increase their energy intake.

## Methods

Animal ethics approval was granted by the University of Canberra’s Committee for Ethics in Animal Experimentation (CEAE 11-22 and CEAE 15-08) and all experiments were performed in accordance with the relevant guidelines and regulations as per the approval. Grassland Earless Dragons were handled and collected under permits from the ACT Government Territory and Municipal Services (Licence to Take LT2012604; Licence to Import LI2011594; LI2012737) and NSW Office of Environment and Heritage (Scientific Licence Section 132c SL100756).

### Study site and climate

We studied the Canberra Grassland Earless Dragon, *Tympanocryptis lineata* (formerly *T. pinguicolla*)^59^, a small agamid now confined to sites near Canberra, Australia (36.31°S, 149.20°E; 580 m a.s.l)^57,59,60^. The Köppen-Geiger climate classification system defines Canberra as “Cfb”: temperate, with no dry season, warm summers (12.5–27.1 °C), and cool winters (0.6–12.2 °C) (mean daily min–max temps; Australian Bureau of Meteorology (BOM) 1939–2008), although summer temperatures have risen in recent years (mean max 30.3 °C January 2008–2022). The maximum monthly summer temperatures on record occurred in the last 5 years (Dec 2019 41.1 °C, Jan 2020 44 °C, Feb 2020 42.7 °C). The air temperatures reported by BOM are recorded at 1.2 m height in a shaded Stevenson box. Ground surface temperatures in the shade (T_shade_) can be up to 20 °C higher than air temperatures (T_a_) on sunny days and ground temperatures in the sun (T_sun_) can reach 70 °C (Fig. 1), similar to other open habitat sites in Australia^61^. Precipitation is relatively consistent throughout the year, with slightly more rainfall in spring (mean monthly 178 mm) and summer (168 mm) than autumn (141 mm) and winter (128 mm) (BOM 1939–2008). Frost is common in winter months.

We captured *T. lineata* at four sites in the Australian Capital Territory (35.3408°S, 149.1814°E.) and adjacent New South Wales (35.3737°S, 149.1940°E) using modified pitfall traps that mimic arthropod burrows the species uses naturally^62^. All sites were within 4 km of each other and at similar elevations (588 to 604 m). The habitat at all sites was natural temperate grassland characterised by *Rytidosperma-Austrostipa* open tussock grassland with no trees or shrubs and a history of livestock grazing with little to no fertilization or pasture improvement^56,63^. Few surface rocks exist and the primary refugia available for *T. lineata* are short, vertical burrows excavated by the Canberra Raspy Cricket (*Cooraboorama canberrae*) and wolf spiders (Lycosoidea).

### Temperature-sensitive radio-telemetry

Following capture, we measured body mass (mean 6.1 ± 0.15 g), snout-vent-length (mean 53.9 ± 0.59 mm), sexed dragons by inspecting for hemipenes, and fitted individuals greater than 4.3 g with an external temperature-sensitive radio transmitter (model BD-2XT, Holohil Systems, Canada or model PIP31, Sirtrack, NZ)^45,46,64^. Transmitters (0.43–0.55 g) were attached to the dorsal base of the tail posterior to the vent opening with the flexible 10–15 cm thin whip antenna positioned to run parallel to the tail^65^ and, with one exception, were retrieved before battery failure. On average, transmitters represented less than 7% of the dragon’s body mass (range 4.7–9.1%; dragon mean mass 6.1 g; range 4.6–9.1 g, n = 44) similar to those used in comparable studies^65,66^, and always < 10% of animal body mass as recommended for small (< 30 g) non-flying animals^47^. Transmitter battery life varied from 5 to 40 d and 30 individual dragons (2012-13 6 F/8 M, 2013-14 7F/9 M) were tracked from 1 to 38 d (mean 14.5 d). Data from two individuals were excluded from analysis as they returned only partial days of data resulting in a final dataset from 28 dragons (see “Data analysis”). Transmitter data was collected using remote receiver/data logging stations comprising a three element Yagi antenna (Sirtrack, NZ) communicating with a receiver data logger (SRX_DL2, Lotek Wireless Inc, Canada). The data loggers were programmed to search for each dragon frequency and record transmitter pulse rates of each transmitter for 90 s every 10 min. The precise location of each dragon was confirmed several times each day using a handheld radio-telemetry device and a short wand antenna.

Temperature-sensitive transmitters contain a thermistor configured so that an increase or decrease in temperature results in a corresponding increase or decrease in pulse rate. Although calibration curves are provided for individual temperature-sensitive transmitters by the manufacturer, we calibrated transmitters in the laboratory immediately before deployment to ensure accuracy. Pulse rates were calibrated in a water bath in the laboratory to an accuracy of 0.1 °C at temperatures from 5 to 45 °C before attachment. Individual transmitter pulse rates were converted to temperatures (T_trans_) using a quadratic polynomial function fitted to calibration data using least squares regression so that each transmitter had a unique curve. The calibration was re-confirmed after transmitters were retrieved^46^.

### Measurement of field microhabitat temperatures

We recorded environmental temperatures in the microhabitats available to *T. lineata* at each of the four study sites during the same period that transmitter temperatures were monitored. At each site, temperature data loggers (Thermochron iButtons®, Model DS1921G,  ± 0.5 °C, Maxim Integrated Inc., USA) were placed in microhabitats previously identified as used by *T. lineata* and in which *T. lineata* had been captured and tracked. iButtons have been used extensively to sample habitat temperatures^45,67,68^ and have been found to reliably record microhabitat thermal variability and predict temperatures experienced by small ectotherms^33,37^. The microhabitats measured were: (1) exposed open ground with vegetation cover less than 2 cm high (T_sun_); (2) shaded open ground (T_shade_); and (3) at the bottom of 15–20 cm deep burrows (T_burrow_). iButtons used to measure open ground temperatures (T_sun_) were wrapped in paper and placed in lengths of copper pipe (24 mm diameter, 55 mm length) that had been spray painted beige and terminated with 22 mm diameter plastic plugs. We used copper pipes to buffer iButtons from direct radiant heat and to record temperatures in full sunlight that would be experienced by *T lineata*. We measured T_shade_ at ground level using iButtons attached to the base of a wooden stake and covered to prevent radiant heating. Burrow temperatures (T_burrow_) were measured using iButtons placed in handmade burrows (25 mm wide, 15–20 cm deep) formed by hammering a length of stainless-steel pipe into the ground. The depth of burrow at which the iButtons were placed approximated the depth of natural burrows used by *T. lineata* in the field (n = 42, mean = 16.6 cm depth, range = 10–27 cm). Burrows were too narrow to accommodate inflexible rigid copper pipes without making the diameter larger than natural burrows. Studies comparing iButtons encapsulated in different materials have found temperatures differed by ~ 1 °C under shaded conditions^69,70^. At each study site and within each microhabitat, we installed at least 3 replicate sets of iButtons. We estimated the temperature in each microhabitat at each study site during each time period as the average of the replicate iButton values. Maximum daily air temperatures (T_air_) were taken at 1.2 m above ground in a Stevenson screen recorded at the Australian Bureau of Meteorology (BOM) Canberra Airport Site 070351. All four study sites were within a 7 km radius of the Canberra airport and at the same altitude.

### Transmitter temperature evaluation

Temperature-sensitive transmitters were used to record skin temperatures (T_skin_) of individuals. Such transmitters have been shown to be a reliable representation of cloacal/ core body temperatures (T_body_) in small ectotherms^33,36,41,71^, and to simultaneously indicate the microclimate temperature experienced by small ectotherms at any given time^33^. The relationship between T_skin_, T_body_, T_trans_, and iButton temperature (T_shade_ and T_sun_) was verified in the laboratory using five female *T. lineata* from a captive colony at the University of Canberra exposed to two treatments: ambient temperatures ranging from 15 to 40 °C in incubators and radiant heating from 20 to 35 °C under a solar heat lamp. Each individual *T. lineata* had a transmitter attached dorsally to the tail base as per the field methodology and two T-type copper-constantan thermocouples (MicroDAQ, Contoocook, NH, USA) connected to a precision digital thermometer (Model HH23A Microprocessor Thermometer, Omega Engineering Inc., Stamford, CT, USA) attached to the dragon using clear surgical tape (Op Site Flexigrid, Smith & Nephew Pty Ltd, Australia). One thermocouple was taped to the dorsal surface of the individual to measure T_skin_ and a second thermocouple inserted several millimetres into the cloacal (T_body_) and taped in position. Only female dragons were used for evaluating T_trans_ to avoid the risk of damage to male hemipenes of this small, sexually monomorphic, endangered species during insertion of a thermocouple into the cloaca.

For comparison of T_trans_, T_skin_, T_body_ and iButton performance in ambient heat, each dragon, with a transmitter, skin and cloacal thermocouples attached, was tested in calibrated incubators at temperatures ranging from 15 to 40 °C for a duration of 5 h with a 5 °C temperature increase each hour. To compare these same temperatures under radiant heat, dragons with transmitter, skin and cloacal thermocouples attached were placed on native grassland soil in a 5 L container with a 100 W mercury-vapour bulb (Zoo Med Laboratories Inc., San Luis Obispo, USA) positioned 260 mm from the surface. iButtons inside copper pipes (24 mm diameter, 55 mm length) were placed on the sediment surface adjacent to dragons. T_trans_, T_skin_,T_body_, and iButton temperatures were recorded every minute for 40 min at a rate of warming of 1 °C per minute from room temperature (20 °C) to a plateau of 30 to 35 °C. Regression analysis was used to compare differences between thermal measures under ambient and radiant heating treatments across the temperature ranges. However, owing to differences in the speed of warming, we only compared T_trans_ to T_sun_ for the final 20–30 min of the test period once both temperatures reached a plateau of 30–35 °C (Mean T_trans_ 34.8 ± 0.13 °C; Mean T_sun_ 33.8 ± 0.06). The time lag in heating of copper pipes from 20 to 30 °C compared to T_trans_ was less than 8 min.

### Determination of *T lineata* panting threshold and critical thermal minimum

All tests for thermal tolerance thresholds took place in an air-conditioned laboratory at 21 °C. To prepare individuals (n = 10; body mass 5.3 to 8.8 g; mean 6.9 ± 1.1 g) for testing, two T-type copper-constantan thermocouples (MicroDAQ) were attached to each dragon using clear surgical tape. One thermocouple was taped to the dorsal surface of the individual and a second thermocouple inserted several millimetres into the cloaca and taped in position. Dorsal (T_skin)_ and cloacal temperatures (T_body_) were recorded every minute.

We used the panting threshold, the body temperature at which dragons started to pant open-mouthed, as our measure of the dragon’s upper thermal tolerance. Individuals were placed in a plastic holding tank containing soil from the native grasslands under a 160 W self-ballasted mercury-vapour bulb and observed until they reached the panting threshold. The dragons were clearly distressed at this point and started to turn over to expose their lighter coloured ventral abdomen to the heat. Their status as an endangered species precluded exposing them to higher, potentially lethal, temperatures.

The critical thermal minimum was determined using the same individual *T. lineata* as used for the CT_max_ experiments. Each individual was placed in a dry 0.5 L plastic container submersed to the rim in ice water at 3.5 to 5 °C. Once T_body_ had reached 16 °C individuals were gently flipped over onto their back and allowed to right themselves. CT_min_ was considered to have occurred when individuals could no longer right themselves and became immobile.

### Respirometry

We measured the resting metabolic rate (RMR) of 12 individual *T. lineata* (6 males, 6 females) from the captive breeding colony at the University of Canberra, Australia using open-flow respirometry to measure oxygen consumption^72,73^. Individuals were housed outdoors for several weeks before RMR measurements were conducted in November 2013. Respirometry was conducted on post-absorptive dragons during their rest phase (night) in darkened temperature-controlled cabinets. Individual body mass (± 0.01 g) was measured at the start and end of each trial and a linear rate of mass loss was assumed for calculation of mass-specific metabolic rate^47^. Oxygen consumption was measured using an oxygen analyzer (FOX, Sable Systems International Inc., USA), placed inside an insulated box in a temperature–controlled room at 19 ± 2 °C. A sub-sampling design was used to keep the rate of flow of the sample air through the analyzer constant (63 ml min^–1^) throughout the measurements. Outside air was pumped through silica gel to remove moisture while rotameters controlled the rate of airflow to the chambers. After passing through the chamber, excurrent air was dried again using silica gel and the flow rate of air was measured using a mass flow meter (Omega FMA-5606, USA). A chamber flow rate of 100 ml min^–1^ was maintained throughout the experiments, which was sufficient to maintain the oxygen content in the excurrent air above 20%. The excurrent air from each chamber (2–3 chambers used) was sampled every 3 mins, followed by 3 mins sampling of a reference channel of dried outside air. Thus, one measurement for each dragon was obtained every 9 to 12 mins^74–76^. Measurements of ambient temperature (T_a_) were taken simultaneously to those of RMR via calibrated T-type thermocouples in the respirometry chambers. Data acquisition and processing were performed using software written by G. Körtner^77^. A respiratory quotient of 0.85 was assumed for all measurements and the rate of oxygen consumption was calculated using Equation 3a of Withers^73^. RMRs were calculated for each individual as the average of the six consecutive lowest VO_2_ values in resting individuals at each T_a_^78^. Chamber temperatures were set to 20, 25, 30, 35 and 38 °C for a minimum of 2 h. Testing of individuals commenced at dusk at the highest temperature setting (38 °C) to be most comparable to temperatures in the outdoor cages during summer and treatment proceeded progressively to each lower temperature over a 15 h period. This was determined to be the best procedure to allow for all measurements to be completed on an individual in one night to minimise handling and stress on important individuals during the breeding season. Only data from the final 75 min of testing once the chamber had reached the designated T_a_ were considered in the analysis.

### Data analysis

Transmitter pulse rates were converted to temperatures (T_trans_) using a quadratic polynomial function fitted to calibration data using least squares regression. Transmitter pulse rates (accuracy 0.1 beats per min) were averaged across each 90 s recording period to obtain a single T_trans_ for each individual every 10 min. Days with recordings that spanned less than 10 h, recordings outside daylight hours, and time periods with ≥ 120 min of missing data were excluded, resulting in a final dataset that comprised data from 28 dragons, 144 days, and 19,735 transmitter temperature observations. Nocturnal scotophase measurements were excluded from analysis as *T. lineata* moved deeper into burrows at night and the transmitter signal was often lost or intermittent with significant data gaps. Values of T_trans_ were matched by time and location (study site) to the mean of the three microclimate temperature replicates for T_sun_, T_shade,_ and T_burrow_ at the respective site. This matching allowed us to determine the most likely microhabitat that individuals occupied at each recording time (the value of T_sun_, T_shade,_ or T_burrow_ that most closely matched T_trans_). Dragons always remained within 100 m radii, and rarely moved more than 50 m radii, from the capture location creating small, uniform study areas. As the temperature differentials between each of these three microclimate types often exceeded 10 °C we could be confident in our inferences of dragon microclimate selection. We used the dragon transmitter and microhabitat temperatures, along with variation over time in the individual transmitter temperatures and signal strength^79^, to classify dragons as either active or inactive at each recording time. We scored an individual as active if T_trans_ was greater than both T_shade_ and T_burrow_ during daylight hours (sunrise to sunset), indicating that the dragon was in the open and not seeking refuge (Fig. 2). Individuals were scored as inactive if T_trans_ was below T_shade_ for at least two consecutive measurements (20 min), implying dragons were in cooler refugia such as burrows, or if at least three consecutive measurements of T_trans_ showed little variation in transmitter temperature (defined as a difference of < 0.5 °C) or signal strength, indicating the dragon was stationary at one location for at least 30 min.

All analyses were conducted in R 4.1.1 (The R Foundation for Statistical Computing, Austria). We classified each day for which we had dragon temperature measurements according to the daily maximum temperature grouped into 5 °C bands (see Fig. 4). Within each band, we calculated the proportion of time for each hour of the day between sunrise and sunset that *T. lineata* were classed as active. We estimated the 95% confidence intervals around these hourly proportions using Wilson’s method for binomial data^80^ as implemented in the R package binom^81^.

We determined the relationship between the proportion of daylight hours that dragons were active and daily maximum temperature (Fig. 5A) by fitting a quadratic regression model to these data using the lm function in R. We determined the relationship between temperature and RMR (Fig. 5B) by fitting a random effects model using the lme4 package^82^, with dragon identity included as a random effect (to account for repeat measurements on the same dragon) and temperature and RMR both log transformed. Mean metabolic rate as a function of daily maximum temperature (Fig. 5C) was calculated by estimating RMR as a function of mean animal temperature (from T_trans_ values and the RMR-temperature relationship shown in Fig. 5B) for each hour of the day, and then calculating overall mean RMR according to daily maximum temperature grouped into 5 °C bands. The non-linear relationship between mean metabolic rate and daily maximum temperature was then estimated using LOESS (locally estimated scatterplot smoothing) with the function loess in R (the red line in Fig. 5C). Mean energetic cost was calculated as mean RMR divided by the proportion of time active and plotted against daily maximum temperature also with a LOESS fit (Fig. 5D).

We downloaded data on daily maximum temperature recorded at Canberra airport from 1941–2020 (downloaded from: http://www.bom.gov.au/climate/averages/tables/cw_070014.shtml) and filtered the data to include only summer months (December, January, February). We calculated the mean daily maximum temperature over these months for each year, and expressed the mean as an anomaly from the mean for the period 1960–1990 (Fig. 6A)—The standard reference period for reporting meteorological data used by the Australian Bureau of Statistics. We estimated the proportion of time that dragons were active during daylight hours for each day during summer from the quadratic relationship with daily maximum temperature shown in Fig. 5A. We then calculated mean proportion of time dragons were active during daylight hours (activity window) for each year expressed as a proportion of the mean for the period 1960–1990 (Fig. 6B). We calculated the mean energetic cost (mean hourly resting metabolic rate/proportion of daylight hours active) for each day during summer from the LOESS relationship shown in Fig. 5D and then calculated the overall mean energetic cost in each year expressed as a proportion of the mean for the period 1960–1990 (Fig. 6C).

## Data Availability

All data and code files related to this manuscript are available at https://zenodo.org/record/7305580#.Y2rEIHZBxaQ (https://doi.org/10.5281/zenodo.7305580).
